# Acetylation- and Methylation-Related Epigenetic Proteins in the Context of Their Targets

**DOI:** 10.3390/genes8080196

**Published:** 2017-08-07

**Authors:** Nasir Javaid, Sangdun Choi

**Affiliations:** Department of Molecular Science and Technology, Ajou University, Suwon 443-749, Korea; nasirjavaid1989@gmail.com

**Keywords:** epigenetics, acetylation, methylation, modifier, epigenetic diseases, drug

## Abstract

The nucleosome surface is covered with multiple modifications that are perpetuated by eight different classes of enzymes. These enzymes modify specific target sites both on DNA and histone proteins, and these modifications have been well identified and termed “epigenetics”. These modifications play critical roles, either by affecting non-histone protein recruitment to chromatin or by disturbing chromatin contacts. Their presence dictates the condensed packaging of DNA and can coordinate the orderly recruitment of various enzyme complexes for DNA manipulation. This genetic modification machinery involves various writers, readers, and erasers that have unique structures, functions, and modes of action. Regarding human disease, studies have mainly focused on the genetic mechanisms; however, alteration in the balance of epigenetic networks can result in major pathologies including mental retardation, chromosome instability syndromes, and various types of cancers. Owing to its critical influence, great potential lies in developing epigenetic therapies. In this regard, this review has highlighted mechanistic and structural interactions of the main epigenetic families with their targets, which will help to identify more efficient and safe drugs against several diseases.

## 1. Introduction

Epigenetics provides a partial description for how cloned animals or monozygotic twins show differences in disease susceptibility despite identical DNA sequences [1,2]. Conrad Waddington used the term “epigenetics” for the first time in 1939 to explain “formation of various phenotypes due to interactions between associated genes and their products” [3]. Later, Arthur Riggs defined the term epigenetics as “study of meiotically and/or mitotically heritable changes in gene function unable to be explained by alterations in DNA sequence” [4]. Presently, this term has been widened to encompass both heritable and transient changes in nature [5]. Here, we have used the modern definition of epigenetics, which is described as involving both transient and heritable alterations in gene expression without any change in the primary sequence of DNA. 

DNA, being a highly charged polymer, requires intense compaction for its nuclear compartmentalization within eukaryotic cells; this is achieved by its association with a set of basic histone proteins to eventually form a highly organized and compact structure called chromatin. In chromatin, the fundamental repeat unit is the nucleosome, consisting of one octamer comprised of four core histone proteins (H2A, H2B, H3, and H4) and 147 bp of DNA twisted around the outer surface of the octamer in two turns. Less is known regarding the molecular basis of the higher order more folded structure of the nucleosome [6]. The degree of this dynamic folding directly affects some important DNA-related functions such as replication, recombination, and transcription. The formation and maintenance of these differentially folded domains is an important question in the understanding of the regulation of biological processes. Approximately three decades ago, most relevant studies were published explaining post-transcriptional histone modifications and their impact on chromatin folding and activity of relevant genes. In particular, chromatin has been divided into two main types depending upon their folding pattern. (1) The loosely folded part of the chromatin is mostly enriched with acetylation marks and called euchromatin, which is the transcriptionally most active region of the DNA; (2) the tightly folded part of the chromatin is mostly enriched with methylation marks and called heterochromatin, which is a transcriptionally less active region of the DNA. The existing chromatin state can be explained by three biochemical mechanisms: ATP dependent SWI2/SNF2-mediated chromatin remodeling, post-translational modifications of histones, and substitution of histone variant. In comparison to heterochromatin, less is known about the generation, maintenance, and inheritance of euchromatin. Euchromatin is widely considered to be the default state of chromatin; recently silence-antagonizing chromatin modifications have been found that endorse the euchromatic state. These modifications involve the substitution of the H2A histone with H2A.Z [7], methylation of K4H3 and K79H3 [8,9], and acetylation of K16H4 [10].

The acetylated euchromatin was first demonstrated by a chromatin immunoprecipitation assay using hyperacetylated histone-recognizing antibodies, revealing that the global localization of acetylated histones is at DNase I-sensitive regions in correlation with transcriptional activation [11,12]. The N-terminal tails of histones with charged lysine residues provide a promising signaling platform due to their environmental exposure outside of the chromatin polymer, which facilitates various interactions with other proteins and complexes for chromatin remodeling [13,14,15]. These interactions change the charge of the histone tails, which eventually weakens the contact between histone and DNA [16]. Acetylation modification can also change the interactions among histones of adjacent nucleosomes [17], as well as between regulatory proteins and histones [14,15]. These modifications alter the structure and folding of the nucleosome, leading to more permissive and open chromatin for transcription (called euchromatin, as mentioned above); however, there is still confusion regarding whether acetylation is an effect or the cause of increased transcription. Mechanistic studies have identified various enzymes responsible for the removal and addition of these epigenetic marks on DNA and histones.

**Figure 1:**
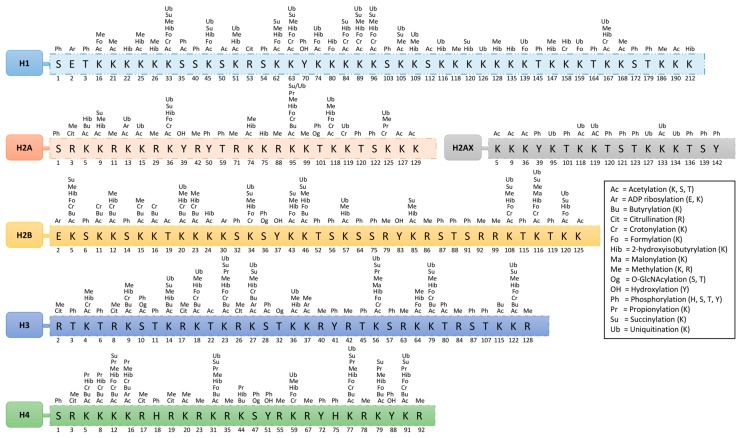
Modification marks of various histones (histone residues are represented by a single-letter abbreviation; the numbers mentioned below the residue depict their relevant position from the N-terminus of protein; modification(s) are abbreviated above the relevant residue; complete name of modification is mentioned in box).

On histones, over 60 different modified residues have been detected by mass spectrometry or using specific antibodies. Some of these modifications are shown in Figure 1 and Table 1. However, there is a huge underestimation of the total number of histone modifications. This becomes more complex when considering the facts that arginine may exist in either a mono- or di-methyl (symmetric or asymmetric) form and that lysine may exist in a mono-, di-, or tri-methyl form within histones of nucleosomes. This variation in modifications is responsible for different functions; however, none of these modifications present at the same time at the same histone site. Their appearance or removal over time depends on the cellular signaling conditions.

These various modifications can be positively regulated by their own histone marks or marks present on the same transcriptional state and vice versa. This kind of interplay can be between the same or different types of modifications. The best-characterized interplay with reference to alteration in gene expression has been reported for two antagonistic groups of epigenetic proteins, *Polycomb* (Pc) and *Trithorax* (Trx) which were first reported for their opposing effects on the *Hox* gene in *Drosophila melanogaster*. Various studies confirm that polycomb repressive complex 2 (PRC2) activity can be inhibited by TrxG methyl modifications at H3K4 and H3K36 on the same histone [18,19,20]. Similarly, activity of some Trx proteins can also be inhibited by Pc group of proteins e.g., PRC1-mediated ubiquitin modification at H2AK119 can inhibit the H3K36 methyltransferases [21]. Different modification marks on the same histone can change the expression state of chromatin, e.g., acetylation of H3K27 is the hallmark of active chromatin while its trimethylation causes silencing of the associated gene. The H3K27 acetylation mark is removed by the NURD (Nucleosome Remodeling Deacetylase) complex, which recruits PRC2 for the tri-methylation of H3K27 at the promoter to repress gene expression level [22]. This phenomenon has been observed in the differentiation of ESCs for the silencing of the previously active genes by the association of NURD with CTBP2 [23]. The NURD complex consists of seven subunits: RbAp48 and RbAp46 (histone binding proteins), HDAC1 and HDAC2 (core histone deacetylase proteins), MTA1, MTA2 or MTA3 (metastasis associated proteins), MBD2 or MBD3 (methyl-CpG-binding domain protein) and CHD3 or CHD4 (chromodomain-helicase-DNA-binding protein). 

Besides the interaction of Pc and Trx group of proteins, there is another phenomenon that controls the expression of genes, called poly(ADP-ribosyl)ation (PARylation). The PARP family includes 17 enzymes but not all of them are active in transferring ADP-ribose. For example, a PARP1 product, poly(ADP ribose), forms a cloud of negative charges on the surface of the modified protein to affect the functionality of associated proteins by electrostatic interactions [24]. The PARylation of TFIIF (transcription factor II F) and TBP (TATA-binding protein) nullify the PIC (pre-initiation complex) formation [25,26]. Similarly, PARylation of the binding sequence of some other transcription factors (like CREB, NFκB, p53, Sp1, YY1) makes them unable to bind at those regions that eventually stop the transcription of relevant genes [27,28,29,30]. On the other hand, PARP1 has also been reported for transcription activation of particular genes by interacting with some other factors like E2F1 and NFκB [31,32]. This diverse role of PARylation in proteins may be due to the involvement of some other modifiers; it has been reported that the binding of NFκB subunit p50 and PARP1 is due to the acetylation of PARP1 by p300 (histone acetyl transferase) [33]. 

**Figure 2:**
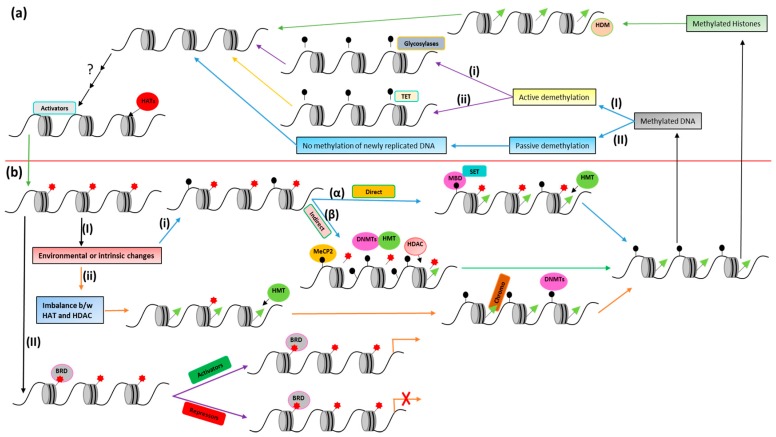
Acetylation and methylation players and their interaction. (**a**) Demethylation and histone acetylation: Methyl marks from chromatin DNA are removed by active demethylation or passive demethylation. (**I**) In active demethylation, (**i**) 5mC (5-methyl cytosine) is converted to thymine and T/G mismatch is repaired by DNA glycosylases (also called direct removal) and (**ii**) the TET (10–11 translocation) enzyme makes various modified forms (5hmC, 5fmC, 5caC, etc.) that are repaired by glycosylases via base excision repair (also called indirect removal). (**II**) In passive demethylation, no methylation of newly replicated DNA happens. Methyl marks from chromatin histones are removed by recruitment of histone demethylases (HDM). Some activators bind to non-methylated chromatin and help in histone acetylation via recruiting histone acetyltransferases (HATs). (**b**) Deacetylation and methylation: (**I**) Due to some environmental or intrinsic changes, (**i**) partial CpG methylation happens and is read by either (α) proteins containing MBD (methyl binding domain) and SET (Su(var)3–9, Enhancer-of-zeste, and Trithorax) domains that recruit histone methyltransferases for direct methylation or (β) MeCP2 (methyl-CpG binding protein2) which recruits HDAC (histone deacetylase), DNMTs (DNA methyltransferases) and HMT (histone methyltransferase) for indirect methylation. (**ii**) Imbalance between HATs and HDACs happens, which causes hypoacetylation and recruitment of HMTs. Histone methylation marks are read by chromodomain-containing proteins (chromo) that recruit DNMTs for CpG methylation. (**II**) Acetylated histone marks are read by bromodomain-containing proteins (BRD): some BRD-containing proteins like SMARCA2, SMARCA4 etc. act like activators of transcription, while others like BAZ1A, BAZ2A etc. act as repressors of transcription.

In this review, we have focused on the acetylation and methylation modification of chromatin. More specifically, structural interactions of proteins associated with these modifications and their targets (DNA and histones) are discussed to better understand the mechanism that will help with the design of therapeutic drugs for various diseases. Moreover, a summary of acetylation and methylation-related players (discussed in this review) and their interaction has been presented in Figure 2. 

## 2. Acetylation-Related Protein Families 

### 2.1. Histone Acetyltransferases (HATs)

Histone acetylation has long been linked with transcriptional activation following its first discovery over 30 years ago [16]. However, no transcription-related activity of HAT was identified until 1996 [51]. Previously, biochemical and genetic studies linked the yeast version of HAT, GCN5, with transcriptional regulation as a coactivator to bridge basal transcription factor and activator protein interactions [52]. This suggested that acetylation of active DNA by DNA-bound activators is due to HATs, and deacetylation of inactive DNA by DNA-bound repressors is due to histone deacetylases [53]. 

For packaging DNA into chromatin, HATs mainly target histone amino-terminal tails where they acetylate lysine residues at ε-amino groups [54]. DNA of 147 bp spools in two turns around a histone octamer (two molecules of each core histone protein, i.e., H4, H3, H2B, H2A) to form a nucleosome. Each histone molecule consists of the following: (1) an amino-terminal domain (highly charged) responsible for histone modification; and (2) a carboxy-terminal domain responsible for nucleosome assembly. Numerous research groups have tried to elucidate acetyl group-transferring mechanisms from acetyl-CoA (Ac-CoA) to histone acceptors by using partially purified fractions or cell extracts in conventional solution enzymatic assays [55]. Based on cellular origin and function, there are two major classes of HATs: (1) cytoplasmic HAT-B catalyzes the acetylation of freshly synthesized histones to move them from the cytoplasm to the nucleus for their deposition on freshly replicated DNA [56]; and (2) nuclear HAT-A catalyzes acetylation events responsible for the transcription [57]. 

**Figure 3:**
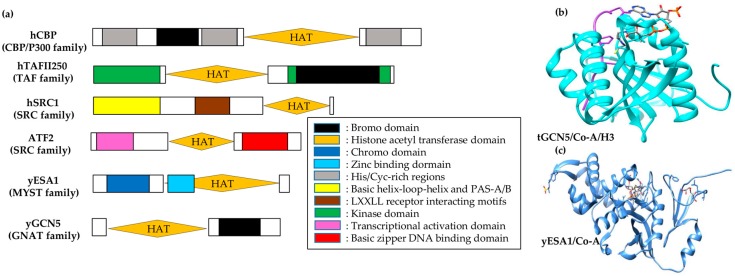
Histone acetyltransferase (HAT) families and complexes. (**a**) Bar diagram of different HAT family members (family name in parentheses) with their associated domains; (**b**) tGCN5/Co-A/histone H3 complex: cyan = tGCN5, purple = histone H3 peptide; (**c**) yESA1/Co-A complex: cornflower blue = yESA1, elemental structure = Co-A.

Depending on the sequence analysis, HATs fall into distinct families with poor to no inter-family but higher intra-family sequence homology [53]. Each HAT family has different substrate-interacting preferences with diverse functional perspectives (see Table 2 and Figure 3a). For instance, the GCN5/PCAF family acetylates H3 at lysine 14 by interacting with transcriptional activators [58]. These proteins also have a bromodomain module at the carboxy-terminal, which acts as a targeting motif for acetyllysine [59]. In contrast, HATs of MYST family (the largest HAT family named after MOZ, Ybf2/Sas3, Sas2 and Tip60) have diverse functions such as dosage compensation in *Drosophila melanogaster* [60], leukogenesis in *Homo sapiens* [61], and cell cycle regulation [62] and gene silencing in yeast [63]. Most MYST proteins, except SAS3, have H4 substrate preference and possess a chromodomain responsible for binding RNA [64].

P300/CBP-associated factor (PCAF), HAT1, and general control nonderepressible 5 (GCN5) belong to a functionally diverse *N*-acetyltransferase superfamily with limited homology within a sequence of four A–D-labeled motifs (15–33 residues), called GNATs (*N*-acetyltransferases related to GCN5) [65]. HAT domain comparison from HAT1 [66], MYST family member yESA1 [67], and the GCN5/PCAF family [68,69] shows structural homology to GNAT proteins for A and D motifs, which make a three-stranded antiparallel β-sheet with an underlying helix. Including another conserved region (loop-β-strand) in GNAT proteins at the immediate C-terminal of A–D motif helix, these collectively comprise a central core domain that is structurally conserved. Among the three HAT families, structural divergence has been observed at the carboxyl- and amino-termini of their core domains. As compared to H3-specific GCN5/PCAF, the structures of H4-specific yHAT1 and yESA1 are more similar to one another. Despite the structural differences between the C- and N-terminals of these families, the loop-α-helix at the C-terminal and the α-helix loop at the N-terminal to the core region superimpose well onto each other.

Structural comparison among HATs shows a conserved core domain that interacts with coenzyme-A (CoA) facilitated by overall Van der Waals and protein backbone interactions by the motif-A residues of GNAT proteins. Structural and functional HAT correlation signifies a catalysis role of the core domain. No *K_m_* change has been identified for either histone or CoA, but a ~360-fold *K*_*ca*t_ decrease was observed in a yGCN5 mutant for E173Q, which signifies the importance of Glu173 in catalysis [69]. Moreover, superimposition of the GCN5/PCAF core domain with that of yHAT1 and yESA1 shows that, despite arising from non-analogous structural elements, Glu255 and Glu338 in yHAT1 and yESA1, respectively, superimpose in 3-D space [67]. Mutagenesis studies of E338Q in yESA1 show consistency for its importance in catalysis. Taken together, the core domain of all three discussed HAT proteins shows conservation in its structure as well as function to bind with CoA and perform catalysis.

The ternary complex structure of tGCN5/CoA/histone H3 has enabled visualization of the mode of GCN5/PCAF binding to the histone [68]. The protein structure shows that a random H3 coil structure (formed by 11 H3 residues centered around H3 Lys14) is bound to a distinct protein cleft in tGCN5 and is flanked by N- and C-terminal segments of protein at opposite ends (Figure 3b). Of these interactions, 75% involve Lys14 and its five immediate C-terminal residues at the H3 backbone. Besides Lys14, Gly13 and Pro16 side chains play a discriminative role in recognizing histone. Ternary complex comparison with apo-tGcn5 and binary tGCN5/CoA structures reveals the structural importance of Ac-CoA in the HAT domain configuration for H3 binding. C- and N-terminal segments are bound by Ac-CoA, which enables histone H3 interactions and widens its effective association. This data shows specificity for histone random coil sequences with a small recognition sequence (G-K-X-P), and indicates an important structural role of Ac-CoA in facilitating this association.

There is an impediment in direct visualization of histone and HAT interactions due to the absence of bound peptides in the yESA1/CoA complex (Figure 3c) [67] and the yHAT1/Ac-CoA complex [66]. However, surface-exposed and conserved residues mapping in HAT families signifies a histone-binding region similar to that of GCN5/PCAF [67]. Correlatively, sequence-conserved regions in core domains of the respective HAT families superimpose well with each other. This superimposition gives structural divergence of C- and N-terminal segments of corresponding proteins. Conserved domains in these HAT families depict histone substrate binding, while divergent sequences modulate specific histone target binding.

### 2.2. Bromodomain-Containing Proteins

HATs are responsible for nucleosomal modifications such as acetylation of histone lysine residues, which are later recognized by protein–protein interacting modules or domains called bromodomains (BRDs). BRDs are evolutionarily conserved domains of 110 amino acids, first discovered in the *brahma* gene of *D. melanogaster* [87]. However, histone-acetylated lysine (Kac) motifs can also be recognized by YEATS domains (Yaf9, ENL, AF9, Taf14, and Sas5) [134], which normally bind to crotonylation-modified lysine residues [135].

Sixty-one BRD modules are encoded by the human proteome and present in 42 different proteins that regulate gene expression in a wide range of activities by recognizing Kac. First, BRDs facilitate the assembly of large protein complexes by acting as scaffolds. Secondly, they can act as transcription coregulators and transcription factors. Lastly, they can perform diverse catalytic functions including roles as ATP-dependent chromatin remodeling complexes, helicases, HATS, and methyltransferases (MTases) (Table 3). BRD proteins show variable and broad expression profiles in various tissues [92]. 

**Figure 4:**
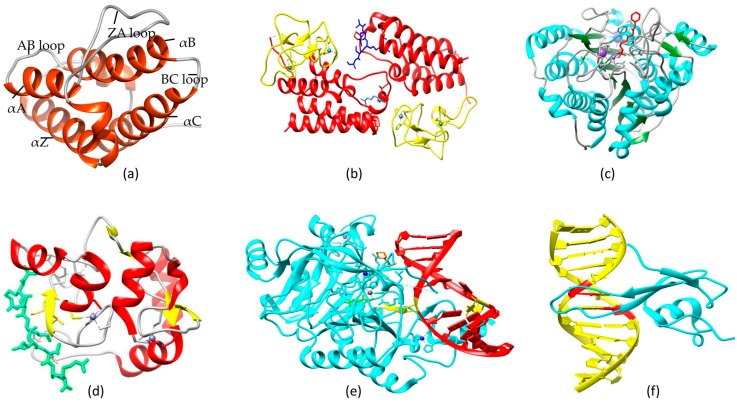
Structure and complexes of epigenetic proteins. (**a**) Structure of SP100 (Speckled 100 kDa): the hydrophobic cavity formed by helices can accommodate histone sequences (PDB ID: 4PTB) (orange = helices, gray = loops); (**b**) Crystal structure of TRIM24 PHD-BRD complexed with H3K27 acetylated peptide (PDB ID: 3O35) (yellow = PHD, red = bromodomain, blue = H3K4ac, cornflower blue = H3K27ac, dark gray = zinc (II) ions (**c**) Crystal structure of HDAC-like protein (Chain A) bound to SAHA (PDB ID: 1ZZ1) (red = SAHA, dark gray ball = Zinc, purple ball = potassium); (**d**) Crystal structure of DNMT3A ADD domain (Chain C) bound to H3 peptide (PDB ID: 4QBQ) (cyan = H3 peptide, dark grey = zinc); (**e**) Crystal structure of TET2 protein in complex with 5hmC (PDB ID: 5DEU) (cyan = TET2, red = DNA, yellow = 2′-deoxy-5-(hydroxymethyl) cytidine 5′-(dihydrogen phosphate), dim gray ball = Fe (III) ion, orange = 2-(*n*-morpholino)-ethanesulfonic acid, green = *n*-oxalylglycine, blue = zinc (II) ion (**f**) Solution structure of the methylated CpG binding domain of human MBD1in complex with methylated DNA (PDB ID: 1IG4) (cyan = binding domain of MBD1, yellow = DNA, red = methylated cytosine).

Multidomain proteins contain BRD modules linked to diverse catalytic and interacting domains via flexible sequences [136]. This specific arrangement allows interactions with various sequence motifs due to conformational flexibility. Some BRDs contain diverse domains such as PHD fingers (plant homeodomain), BAH domains (bromo-adjacent homology), and PWWP domains (Pro-Trp-Trp-Pro), which enable them to interact with various proteins to participate in various biological processes as mentioned in Table 3. More than a decade ago, the distinctive architecture of the BRD module was structurally characterized [59,96,97] as having four α-helices (αB, αC, αZ, and αA) that are linked together by two divergent loops (BC and ZA) (Figure 4a). This module contains fold-stabilizing conserved residues including a PxY motif at the C-terminal of the ZA loop and a Tyr residue in the AB loop forming a salt bridge typically to the Asp residue on αB. Kac docking is facilitated by the conserved Asn residue at the N-terminal of the BC loop, while interactions with the acetylated peptide backbone are initiated by a large charged interface provided by the surface surrounding the Kac-binding pocket. Instead of the usually conserved Asn residue, some BRDs contain a Tyr (as in SP100, SP110, and SP140) or Thr residue (as in BRWD3); however, evidence to link these BRDs with Kac-binding and their capability to recognize specific modifications has not been found. Collectively, a neutralized Kac side chain is accommodated within a small hydrophobic pocket of the BRD module formed by its four α-helices, while the charged surface of the BRD surrounding the Kac-binding site facilitates the entire binding of the acetylated peptide. There are eight families of BRD modules in humans determined by sequence similarity and structural topology [136]. There is dramatic charge variation on the surface of BRD modules: some are highly positive, unlike others, and do not target positively charged Lys and Arg residues carrying acetylated histone peptides. Because of surface charge variation and wide expression variation, it can be speculated that BRD modules may also interact with many other acetylated non-histone proteins. 

Four α-helices of BRD modules form a hydrophobic cavity, which accommodates the neutralized acetylated lysine side chain of the histone peptide sequence [59,96,97]. The conserved mode of binding has been evaluated by analyzing the crystal structure of BRD modules in complex with histone peptide (acetylated), in which one Kac residue inserts into the Kac-binding cavity in BRD and starts the interaction with water molecules (present in cavity) and conserved Asn residues. The orientation of the bound peptide can show dramatic variation depending upon the adjacency of particular domains to the BRD module. For example, proteins that harbor only a single BRD bind histone peptides in such a way that the peptide N-terminus resides at the back-side of the pocket; the peptide inserts itself between the BC and ZA loops and aligns above the Kac-binding cavity with one exit vector over the ZA loop. Notably, this arrangement is changed by the presence of other modular domains. For example, the chromatin regulator TRIM24 (also recognized as TIF1α) engages N-terminal H3K4 by recognition initiated by a PHD finger, while the adjacent BRD module, linked by a flexible loop to the PHD finger, binds to H3K23ac on the same histone protein (Figure 4b) [100]. Similar PHD-BRD domain organization in nucleosome remodeling factor complex subunit, called BPTF, facilitates its binding to H3K4me2 and H3K4me3 by the PHD domain [137], which then allows specific BRD binding to H4K16ac present in trans within the same nucleosome unlike other H4 acetylations [98]. This multivalency in interactions reveals the peptide orientation: for example, the peptide C-terminus is aligned between BC and ZA loops of BRD, complexed between the TRIM33 PHD-BRD cassette and the H3 peptide containing K18ac and K9me3 [138]. The presence of tandem BRDs bound together by a short rigid linker, as found in TAF1, facilitates H4K12ac and H4K5ac binding, eventually increasing the specificity for the histone H4 tails that have multiple acetylation [96]. Joining of two BRD modules by a long flexible linker, as in the BET family, provides conformational plasticity to the structure, which enables it to simultaneously recognize distant acetylated lysine residues present within the same or different proteins [101,102]. In the absence of these multi-domain arrangements, the mode of binding to single acetylated lysine residues inside histone tails appears to be comparatively well conserved among various BRD modules, with little difference in peptide topology implanted in the grove between the ZA and BC loop regions.

Some BRDs have the ability to recognize double Kac histone marks and to bind H4 peptides, having K8ac and K5ac [99]. This example is related to the BRDT protein, in which H4K5ac directly binds to a conserved Asn residue, while H4K8ac inserts into the binding cavity of Kac, interacts with H4K5ac, and has water-facilitated interactions with the BRDT protein. Recognition of double acetylated lysine marks on histones by a single BRD is a common shared feature of BET family members as the BRD domain at the N-terminal of BRD4 complexed with H4 peptide also shows such an interaction with two Kac residues [136]. 

### 2.3. Histone Deacetylases (HDACs)

Many tumor suppressors and oncogenes (e.g., *Rb* and *Mad*) are associated with aberrant HDAC activity, resulting in serious consequences [139]. For example, fusion of the retinoic acid receptor α gene and the promyelocytic leukemia (*PML*) gene in acute promyelocytic leukemia produces an oncoprotein responsible for the recruitment of HDACs to suppress transcription of particular genes, which inhibits cancer cell differentiation and enables their unlimited proliferation [140]. Similar outcomes have been observed for AML1-ETO fusion, PLZF-retinoic acid receptor α fusion, and solid malignancies involving the Myc/Mad/Max signaling pathway [141,142,143,144]. 

There are two HDAC protein families: the classical HDAC family and the recently discovered SIR2 family (NAD^+^-dependent). The classical family has two phylogenetic classes, named class I and class II [145]. Class I members (HDAC 1–3 and 8) have resemblance to RPD3 (yeast transcriptional regulator), while class II members (HDAC 4–7, 9, and 10) have resemblance to another yeast deacetylase, HDA1 [145]. HDAC11 is most relevant to class I, but has not yet been placed in any class because of overall sequence variation [146]. Class II HDACs are considered to be involved in developmental processes and cellular differentiation due to their specifically restricted expression behavior, unlike those of class I [147,148]. More details on class members are shown in Table 4. 

The action mechanism of HDAC enzymes involves acetyl group removal from nucleosome-forming histones. Hypoacetylation causes tighter association between DNA and nucleosomes due to a decrease in the space between them, which eventually represses transcription due to lower accessibility of transcription factors to that region [156]. The HDAC catalytic domain consists of almost 390 amino acids and has a conserved amino acid set. Its active site has a wider bottom containing a tubular pocket [157]. Two neighboring histidine residues, two aspartate residues (present at around a 30-residue distance from histidines and separated from each other by approx. six residues), and one tyrosine residue (123 residues downstream of aspartate residues) collectively form a charge-relay system that removes an acetyl group from the target site (Figure 4c) [147,157]. Zn^2+^ is the essential component of this relay system and is bound to the Zn-binding site situated at the bottom of the pocket. Some other cofactors are also required for proper activity of HDAC, thus most of the recombinant enzymes are inactive without such cofactors. Zn^2+^ displacement is a promising target for most of the HDAC inhibitors (HDACi), while TSA (Trichostatin A) is a good option as a reversible HDACi and has a low nanomolar IC_50_ value due to its 5-carbon atom phenyl group linker and hydroxamic acid group, which collectively allow it to fit into the HDAC active site [158].

Instead of working alone, HDACs form a repressor complex involving other proteins with facilitating functions such as chromatin remodeling, corepression, and recruitment. DNA itself sends important signals for the initiation of repression. Methylated CpG binding domain-containing proteins, methylated CpG-binding proteins, and MTases recruit HDAC complexes at CpG islands (DNA stretch of methylated cytosine residues at 5′ end of guanosine nucleotides). Epigenetic gene silencing, such as X-chromosome inactivation and imprinting, is mainly based on methyl groups such as those in CpG islands, and it seems that HDACs are the only enzymes responsible for this silencing. However, this is not the case as target gene expression is not always restored by inhibiting HDAC activity [159]. In addition to histones, some other proteins including MyoD, α-tubulin, ESF (pre-rRNA processing protein ESF), and p53 are also deacetylated by HDACs, which indicates their functional complexity in many cellular processes [160,161]. 

## 3. Methylation-Related Protein Families

### 3.1. Methyltransferases

**Figure 5:**
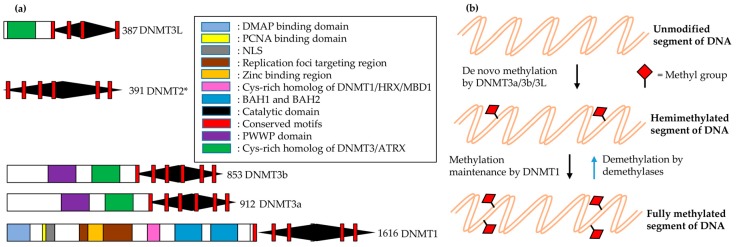
Members of mammalian DNA methyltransferase (DNMT) families. (**a**) Structural comparison of all mammalian DNMTs; (**b**) function of each member for DNA methylation: initial CpG methylation (de novo) is established by the DNMT3 family, while it is maintained by another DNMT family (DNMT1). * DNMT2 is an example of divergent evolution and methylates the tRNA^Asp^ anticodon loop at Cys38.

In most vertebrates, including mammals, C5 of the cytosine within CpG dinucleotides is the main site for DNA methylation. This methylation involves the following major steps: methyltransferase (MTase) binds to target DNA; everts target nucleotides from the double helix (base flipping); attacks C6 of cytosine by a conserved cysteine nucleophilic residue; transfers a methyl group to activated cytosine C5 from S-adenosyl methionine (AdoMet); and is finally released from the complex. Histone modifications and methylation modulate the chromatin structure, which eventually controls chromatin-dependent processes such as gene expression [162]. Mammals have two distinct families of DNA nucleotide methyltransferases (DNMTs), which collectively have four members for which the structural and functional information is provided in Figure 5. 

The DNA replication fork is hemimethylated (presumably for repairing damaged sites) by DNMT1 when it is targeted by SRA protein/ubiquitin ligase ICBP90 (human)-Np95 (mouse) [163]. It seems that the transition of DNMT1 to the active state involves major conformational changes, which include interactions between catalytic domains and the amino-terminal [164] and/or Ser515 phosphorylation [165].

The germ cell-specific knockouts for DNMT3L and DNMT3 are indistinguishable in terms of DNA methylation pattern in germ cells and retrotransposon dispersion, which indicates the requirement of both for the imprinting of germ cell loci [166,167]. Moreover, DNMT3L enhances de novo methylation by both DNMT3a and DNMT3b due to coimmunoprecipitation and colocalization with these DNMTs [168]. The minimal required region for successful interaction between DNMT3L and DNMT3a or DNMT3b lies in their respective C-terminal domains [169], which show a characteristic fold similar to Class I AdoMet-dependent MTases [170]. However, the methylation reaction product S-adenosyl-L-homocysteine (AdoHcy) was not found in DNMT3L-C, unlike DNMT3a-C, which is consistent with the finding that DNMT3a-C is catalytic in the form of a complex, while DNMT3L alone is not capable of being active and binding to AdoMet [171]. The overall length of the DNMT3L-C/DNMT3a-C complex is approximately 16 nm long (more than the diameter (11 nm) of a core nucleosome). Two monomers of each member comprise this complex and form a tetramer with one 3a–3a interface and two 3L–3a interfaces (3L–3a–3a–3L). Substitution of key residues at these interfaces demolishes the enzymatic activity, which highlights the presence of both interfaces for proper catalysis [172]. The conformation of an active site loop in DNMT3a is stabilized by the interaction of its C-terminal residues (G718–L719–Y720) with DNMT3L. These interactions may explain the role of DNMT3L in DNMT3a activity stimulation [169,173]. Moreover, the intrinsic activity shown by DNMT3a–3L heterodimers is higher than that of DNMT3a–3a homooligomers owing to the positive impact of DNMT3L on the catalytically competent closed conformation of the active-site loop of DNMT3a by reducing the available conformational space for that loop [172].

The smallest DNA-binding domain amongst all known DNA MTases is present in DNMT3a and DNMT3b (it is almost absent in DNMT3L), and consists of ~50 residues as compared to 85 in M.HhaI (bacterial GCGC MTases) [174]. However, the DNA binding surface doubles in size by bringing together two active sites via dimerization of the 3a–3a interface. A contiguous DNA is formed via the connection of two DNA segments in such a way that the two active sites are positioned in the major groove of DNA around 40 Å apart. This model reveals that two CpGs can be methylated simultaneously in one binding event of dimeric DNMT3a if they are present at a separation distance of one helical turn. DNMT3a activity on long DNA substrates shows periodicity, revealing a correlation of methylated CpG sites (8–10 base pairs apart from each other), which could be explained by a structural docked model of oligomeric DNMT3a to DNA [172]. Twelve maternally imprinted genes in the mouse showed similar periodicity for CpG site frequency in differentially methylated regions [172].

So far, the best-studied histone mark associated with DNA methylation is H3K4me0 (unmethylated histone-3 Lys4). Genome-wide analysis showed an inverse correlation between DNA and H3K4 methylation, i.e., DNA can be protected from de novo methylation by H3K4 methylation [175]. Indeed, DNMT3L can only interact with unmethylated H3K4 via its PHD-like domain [176]. DNMT3L recruits DNMT3a2 (Dnmt3a isoform specific to germ cells) to nucleosomes, having unmethylated H3K4 for de novo methylation [172,176]. This hypothesis is supported by an experiment in which mouse KDM1B (H3K4 demethylase) knockout resulted in increased H3K4 methylation and abolished DNA methylation at oocyte imprinted genes [177]. These findings reveal that H3K4 demethylation is acute for DNA de novo methylation of a few imprinted genes of germ cells.

Interestingly, recent structural and biochemical studies have shown that the DNMT3a PHD (also named ADD) domain can directly interact with H3K4me0 without any accessory proteins in vitro (Figure 4d) [178]. Moreover, the PWWP domain of DNMT3a was observed to interact specifically with H3K36me3 (H3 with trimethylated Lys36) in vitro [179]. DNMT3a2 activity at chromatin-bound DNA is increased by both of these interactions [180]. Possibly, DNMT3a identifies particular modifications at histones following methylation of associated DNA, which is consistent with recent genome-wide studies. For example, active gene bodies contain the H3K36me3 modification [181], which has positive correlation with DNA methylation [181]. In somatic cells, strong DNMT3a/3b interaction with nucleosomes has been observed, but it does not involve DNMT3a/3b binding to histone H3, and the presence of any DNMT3a/3b-interacting protein (e.g., HP1α and EZH2) has not been observed [182].

Contrary to the above, lysine, arginine, and histidine histone residues have been reported to be methylated by histone methyltransferases (HMTs); the latter is the least known, unlike the other two. Lysine methylation activity is shown by enzymes as having a conserved 140-amino acid long SET domain (suppressor of variegation, enhancer of zeste, trithorax), except DOT1L. Around 48 SET-containing proteins have been reported to be encoded by the human genome. Recruitment of lysine-methylating enzymes at histones is assisted by specific DNA sequences, e.g., TREs (trithorax group response elements) and PREs (polycomb group response group elements), which recruit the respective proteins for H3K4 and H3K27 methylation, respectively. Arginine residues are methylated by different sets of proteins, called PRMTs (protein arginine methyltransferases), which methylate guanidine nitrogen at arginine residues using S-adenosyl-L-methionine (SAM) as a methyl group donor. PRMTs have a conserved catalytic core and a variable region at their C- and N-terminals. Asymmetric and symmetric dimethylation of arginine residues is carried out by type I and type II HMTs, respectively (reviewed in [183,184]; Table 5). 

### 3.2. Demethylases

There are two mechanisms for DNA demethylation, i.e., passive (no methylation of the newly synthesized DNA strand during replication) and active (replication-independent removal of methylation mark(s)). The former occurs during development in mammals (e.g., during the pre-implant growth period in the maternal genome) [198], and it has been revealed that DNA hypomethylation can be achieved by DNMT1 inhibition [199]. Here, we will mainly be focusing on active DNA demethylation. A considerable body of results supports active genome-wide demethylation in primordial germ cells (PGCs) [200] and zygotes [198], as well as active locus-specific demethylation in somatic cells including T-lymphocytes [201] and neurons [202]. Different mechanisms for the enzyme-mediated removal of 5mC (5′ methyl cytosine) 5-methyl groups, 5mC bases, or 5mC nucleotides are proposed; however, more than one mechanism may be involved in this process. For example, global demethylation may have a different mechanism to that of locus-specific demethylation. Discovery of 5-hydroxymethylcytosine (5hmC) in the mammalian genome has opened new research avenues to understand mechanisms of active demethylation. 

Only in plants, direct 5mC base removal by 5mC-specific glycosylases has been observed [203]. Regarding 5mC preference in double-stranded DNA, four members of the 5mC DNA glycosylase family (DME, DML2, DML3, and ROS1) have been identified in *Arabidopsis*, with strong genetic and biochemical evidence for particular genes in active demethylation [203]. ROS1 is actually a proto-oncogene tyrosine-protein kinase ROS that plays a role in regionalization of the proximal epididymal epithelium and epithelial cell differentiation. For instance, the bifunctional glycosylase ROS1 shows apyrimidinic/apurinic activity, i.e., it eliminates the target base followed by cleavage of the abasic site, which generates a nick (later repaired rapidly) [203]. This process is similar to that present in mammals for mismatch repair with the elimination of alkylated bases, known as base excision repair (BER). Evidence reveals a role of BER in mammals for active demethylation of target, but the initiation mechanism and enzymes may be dissimilar to those of *Arabidopsis*.

Until now, no homolog of the DME/ROS1 glycosylase family has been identified in mammals; however, fragile glycosylase activity at 5mC has been observed for TDG (thymine DNA glycosylase) and MBD4 (methyl-CpG-binding domain protein 4) [204]. Both of these show 30–40 times lower glycosylase activity on 5mC as compared to T-G mismatch [204], which sheds doubt on their 5mC DNA glycosylase nature [205]. Consistently, zygotic paternal genome global demethylation does not require MBD4, and *mbd4* knockout mice show fertility and viability [206]. However, the locus-specific activity of MBD4 as a 5mC DNA glycosylase cannot be ruled out as hormone-induced MBD4 phosphorylation leads to active demethylation at the promoter region of the *CYP27B1* gene via stimulation of its glycosylase activity [207]. 

Other active DNA methylation-related proposed mechanisms also involve BER, but after the 5mC base has been modified. The leading mechanism is conversion of 5mC to thymine by its deamination following the removal of the resulting T-G mismatch via BER machinery. AID (activation induced cytosine deaminase) and APOBEC1 (apolipoprotein B mRNA editing enzyme, catalytic polypeptide 1) can generate T-G mismatches after 5mC deamination, but both preferentially target single-stranded DNA [208]. Expression of both enzymes has been observed in mouse oocytes, and AID has also been observed in PDCs (plasmacytoid dendritic cells), which suggests a potential role of these enzymes in global DNA methylation [208]. These results led to the hypothesis that mammalian demethylation can occur by deamination of 5mC, with subsequent BER started by T-G mismatch glycosylases, such as TDG and MBD4 [208]. This hypothesis is supported by experiments in zebrafish embryos that overexpress both MBD4 and AID; however, neither alone resulted in DNA demethylation [209]. Moreover, some studies revealed that AID has a role in active mammalian demethylation [210]. Using heterokaryons formed by fusing human fibroblasts with mouse ES cells, Bhutani et al. [211] revealed that AID is required for active demethylation of NANOG and OCT4 promoters during fibroblast genome reprogramming by cell fusion. Another group performed a study in PGCs for wild type and *Aid* knockout mice and observed wide DNA methylation of the genome [210]. As compared to wild-type PGCs, *Aid^−/−^* mice showed a higher methylation level in the whole genome [210]. However, in the absence of AID, significant demethylation still occurred as a low methylation level was detected in *Aid^−/−^* PGCs [210] without any developmental defects in mice that were fertile [212], suggesting that there are some other factors in PGCs that participate in global demethylation.

In addition to BER, the involvement of NER (nucleotide excision repair), another DNA repair pathway, has also been examined in relation to active demethylation. Barreto et al. [213] used an expression cloning approach to prove that protein factor GADD45a in mammalian cells can promote active global demethylation by involving the NER pathway owing to DNA synthesis and XPG (NER endonuclease), which binds directly to GADD45a. However, another study could not confirm this finding [214], and neither a global nor a locus-specific increase in methylation was seen in *Gadd45a^−/−^* mice [215]. However, a locus-specific DNA demethylation role of GADD45 family proteins has been supported by some studies [202,216]. It has been observed for the rRNA gene promoter that active demethylation happens by NER machinery and GADD45a [216]. Another GADD45 family member, GADD45b, has been observed for locus-specific demethylation at regulatory regions of *Fgf1* and *Bdnf* genes, which are involved in neurogenesis due to neuronal activity in mature hippocampal neurons [202].

The discovery of 5hmC and its associated enzymes in mammalian cells has opened up new avenues for demethylation studies. 5mC was considered to be the only naturally modified mammalian DNA base until the discovery of 5hmC in ES (embryonic stem) cells [217] and mouse Purkinje neurons [218]. By searching for trypanosome thymidine hydroxylase homologs in mammals, researchers found three ten–eleven translocation (TET) family proteins in humans (TET1, TET2, and TET3) and revealed that TET1 can convert 5mC to 5hmC in cultured cells and in vitro [217]. Similar reactions can be performed by all three TET proteins in the mouse; however, TET1 shows a role in self-renewal of ES cells and inner cell mass specification [219]. The crystal structure of TET2 and 5hmC complex is shown in Figure 4e. TET1 retains the hypomethylated state of the *Nanog* promoter in mouse ES cells, suggesting its role in DNA methylation regulation [219]. One postulated mechanism revealed BER involvement initiated by 5hmC-specific DNA glycosylase [217]. It is notable that the calf thymus has been reported as having 5hmC-associated glycosylase activity [220]. 

H3K4 and H3K9 methylation marks are removed by lysine-specific demethylases, LSD1 and LSD2, through an FAD-dependent amine oxidation reaction [221]. Meanwhile, H4K20, H3K36, H3K27, H3K9, or H3K4 can be demethylated by another family of histone demethylases, the JMJD (Jumonji C domain-containing) family, which contains a JmjC domain (150 amino acids) [222]. Less is known regarding methylation mark removal from arginine: a new pathway for arginine methylation reversion in mammalian cells has been studied. This involves the conversion of methylarginine to citrulline by removing its methyl group, a process known as deamination, at specific sites on H3 and H4 tails with the help of enzyme PADI4 (peptidyl arginine deiminase 4) [50,223]. Moreover, H3R3me2 and H3R2me2 demethylation have been reported for the first time with JMJD6/PSR/PTDSR (phosphatidylserine receptor) [224]. However, different studies have questioned this activity of JMJD6 [225,226]. Further study is required to validate the arginine demethylase activity of JMJD6. 

### 3.3. Methyl Binding Proteins 

Two mechanisms have been identified for the repression of gene expression via DNA methylation. The first direct mechanism involves DNA methylation-mediated alterations in binding sites of transcription factors such as CREB and E2F, which eventually prevents transcription activation [227,228]. Another elaborative mechanism involves the recruitment of methyl-CpG binding proteins (associated with different chromatin modifiers), which creates a repressive chromatin environment [229]. These proteins make a connection between chromatin modification and DNA methylation via reading and interpreting epigenetic signals. Proteins of the methyl-CpG binding domain (MBD) family have been widely studied, and their characterization reveals their various functions (Table 6). Mutations in methyl-CpG binding protein 2 (MeCP2), an MBD family founder, result in X-linked neurodevelopmental disorder and Rett syndrome (RTT) [230]. Other proteins of the MBD family bind to irrationally hypermethylated promoters in different cancer cell lines of human origin [231]. Initially, MBD was identified as the minimal part of MECP2 required for methylated DNA binding [232], and the homology of its amino acid sequence with other proteins led to the discovery of MBD1, MBD2, MBD3, and MBD4 [233]. The solution structure of the methylated CpG binding domain of human MBD1 in complex with methylated DNA is shown in Figure 4f. MBD1, MBD2, and MeCP2 have been reported to contain a non-conserved domain responsible for transcriptional repression. Apart from the MBD domain, MBD1 has a CxxC3 zinc finger domain for DNA binding [234], which has sequence similarity with the CxxC domain of DNMT1 [235]. Preferably, methylated but not unmethylated DNA is recognized by all MBD proteins except mammalian MBD3 [236] and MBD3 LF (amphibian MBD3 long form), which is unable to specifically recognize DNA methylation due to an insertion in the MBD region [237]. Generally, depending on the sequence context, MBD proteins show 3- to 10-fold higher affinities for methylated DNA as compared to unmethylated DNA [238]. In vitro experiments for binding site selection showed that an A/T-rich sequence adjacent to the site of CpG methylation is required by human MeCP2 [239]. Moreover, some other methylated DNA binding proteins aside from the MBD family have also been identified [163,240,241,242,243,244,245]. 

## 4. Epigenetics and Human Diseases

Environmental chemical exposure, inherited genetic polymorphisms, and changes in diet lead to fluctuation in patterns of DNA methylation [276]. Diet-acquired methyl groups are transferred to DNA by methionine and folate pathways [277]. Serious clinical consequences including atherosclerosis, cancer, and neural tube defects may occur due to alterations in DNA methylation by consuming a diet with low methionine, folate, or selenium [278,279]. Such nutrient imbalance in the diet cause genetic instability (leading to chromosome rearrangement) and hypomethylation (leading to unfit gene expression) [279]. For example, in vitro models of atherosclerosis revealed global hypomethylation and hyperhomocysteinemia supporting the hypothesis that alterations in patterns of global methylation are attributes of this disease at early stages [278], while hyperproliferation at advanced stages may further enhance hypomethylation of DNA and alterations in gene expression [280].

Environmental agents such as aromatic hydrocarbons (e.g., benzopyrene) and metals (e.g., arsenic) can also cause modification of cellular metabolism or destabilization of the genome, or both [280]. These agents are present in fossil fuel emissions, cigarette smoke, contaminated drinking water, and occupational chemicals [281]. Diet or environmental toxin sensitivity depends on previously existing genetic variants capable of challenging methylation-related metabolism and predisposing a person to changes at the epigenetic level. Some studies have linked the methylenetetrahydrofolate reductase gene (*MTHFR*) to altered patterns of DNA methylation in response to hormone replacement, alcohol consumption, and diet, which eventually result in a high incidence of colorectal and breast cancer in specific populations. For example, in premenopausal women, a common polymorphism in MTHFR 677CT increases breast cancer by up to 3-fold [282]. Other studies revealed that by hormone replacement therapy in postmenopausal women of the 677TT genotype, a 40% decrease in breast cancer risk was observed, which is probably owing to the limiting nucleic acid precursor availability for hyperproliferating cells [283]. Such examples highlight the complex interplay among disease-enhancing risk factors including epigenetics, genetic individuality (nature), and environment (nurture).

Multicellular organisms require specialized mechanisms for heritable gene silencing patterns, and mutation in these genes alter global epigenetic modification profiles resulting in many somatically acquired or inherited diseases. Interestingly, most of these abnormalities cause learning disabilities and chromosomal alterations. For example, *atrx* gene mutation changes the methylation pattern of ribosomal DNA, subtelomeric repeats, and Y-specific repeats. Fragile X syndrome occurs by de novo expansion and methylation of the CGG repeat in the 5′-untranslated region of the *FMR1* gene, which creates a prominent “fragile” site on the X chromosome by rendering it silent under certain conditions. Globally, mutations in the *dnmt3b* gene affect establishment of patterns of DNA methylation resulting in immunodeficiency, centromeric instability, facial anomalies (ICF) syndrome [284]. These findings revealed a primary importance of epigenetic modifications in determining chromosomal architecture. 

Faulty genome imprinting (described as parent-specific monoallelic gene expression) causes many inherited syndromes such as Beckwith–Wiedemann syndrome (BWS), Prader–Willi syndrome, and Angelman syndrome. In these syndromes, deletion or uniparental disomy (UPD) cause the absence of a maternal or paternal imprinted gene copy or imprinted gene deregulation, which results in abnormal phenotype. For example, an imprinted gene cluster at 11p15.5 participates in BWS pathology: in most cases of BWS, methylation loss in control regions of imprinting causes its deregulation and either silencing (e.g., CDKN1C) or biallelic expression (e.g., IGF2) of associated imprinted regions. 

Particular interest has been sparked by the revelation that MeCP2 germ line mutation causes Rett syndrome [230]. Methylcytosine residues are bound by MeCP2 [285], and disease progression is due to derepression of normally DNA methylation-mediated repressed genes. For this, no direct evidence has been found since gene derepression at a global level has not been observed in MeCP2 mutated human cells [285]. Nonetheless, MeCP2 plays a key role in controlling neuronal gene activity, causing Rett syndrome [286]. 

An interesting recent discovery revealed that antisense RNA transcription causes methylation and silencing of the α-globin gene in thalassemia patients [287]. Conventional diagnostic methods have failed to identify many other such diseases that occur due to improper gene silencing. This thalassemia case might be the tip of the iceberg, indicating that several other diseases might be caused by epigenetic silencing due to inappropriate genomic rearrangements. 

Epigenetic changes are also responsible for many kinds of cancers. For example, the MutL homolog-1 (MLH1)-encoding gene is methylated and silenced with a phenotype of microsatellite instability in a high number of sporadic colorectal cancer patients [288]. Thus, genetic instability is directly linked to genetic silencing. Sometimes, promoter-associated MLH1 methylation is found in normal tissues (e.g., spermatozoa) along with tumor cells, and these germline-linked “epimutations” predispose these patients to multiple cancers [289]. De novo methylation of the promoter region of a gene causes disruption of the associated pathways that lead to cancer [290]. Epigenetic silencing is considered the third pathway correlating with Knudson’s hypothesis that tumor-suppressor gene silencing requires two hits [291].

Different chromatin-modifying enzymes are causative agents for various hemopathologies. For example, HMTs and acetyltransferases cause chromosomal translocation and the expression of fusion proteins at target sites in leukemia patients [292]. In acute cases of promyelocytic leukemia, the fused oncogenic protein PML-RARα (promyelocytic leukemia-retinoic acid receptor-α) represses hematopoietic cell differentiation genes via recruiting an HDAC [293]. Similarly, in acute cases of myeloid leukemia, the fusion protein AML1-ETO inhibits myeloid development via recruiting the NCOR–SIN3–HDAC1 complex [294]. 

The significance of accurate chromatin composition is further supported by the disease involvement of ATP-dependent chromatin remodeling multisubunit complexes, which are capable of transcriptional regulation via shifting and moving nucleosomes. Various cancers involve many members of the SWI–SNF complex, which is a highly conserved chromatin remodeler [295]. For example, SNF5 loss is observed in pediatric cancers, and mutation in BRG1 and BRM ATPase subunits is seen in various primary tumors and cancer cell lines; this is linked with poor prognosis in non-small cell lung cancer patients [295]. 

## 5. Conclusions

Many human diseases are linked to inappropriate epigenetic modifications; thus, researchers are attempting to identify relevant drugs to reverse these modifications. For example, various inhibitors against undesired HDACs (HDACi) have been analyzed in animal experiments, normal healthy cells, and clinical trials with no or few side effects within a therapeutic range [296,297,298,299,300,301]. Similar to acetylation, many methylation inhibitors including 5-azacytidine, 5-aza-2-deoxycytidine, zebularine, and procainamide have been discovered to be effective against improper methylation [199,302,303,304]. A short list of drugs under clinical trials against HDACs and methylation is given in Table 7. Each epigenetic family has its own particular structure and functional interacting domain; however, there is still the possibility that some may have the same interacting pocket conformation, and designing a drug against these common pockets, to stop more than one member of different families, would be a worthy strategy. More in-depth structural and mechanistic studies between epigenetic proteins and their target DNA or histones may help to discover such universal drugs, which will be more economical from a commercial point of view.

## Figures and Tables

**Table 1:** Histone target(s) of various modifications and their associated functions.

Modification (Short Form)	Function	Target Residue/s	Reference
Methylation (me)	Repair, transcription	K-me1, K-me2, K-me3	[34,35]
	Transcription	R-me1, R-me2a, R-me2s	[36]
Acetylation (ac)	Condensation, repair, transcription, replication	K-ac	[37,38,39]
Phosphorylation (ph)	Repair, transcription, condensation	T-ph, S-ph	[40,41]
Sumoylation (su)	Transcription, repair	K-su	[42,43]
Ubiquitylation (ub)	Repair, transcription	K-ub	[44,45]
ADP ribosylation (ar)	Transcription, repair, replication	E-ar	[46,47,48]
Proline isomerization	Transcription	P-cis > P-trans	[49]
Deimination	Transcription	R > Cit	[50]

Abbreviations: a = asymmetric; Cit = citrulline; E = glutamic acid; K = lysine; P = proline; R = arginine; S = serine; s = symmetric, T = threonine.

**Table 2:** HAT family members and their properties.

HAT Family	Proteins	Enzyme	Organism	Orthologs	Substrate	Function	References
GNAT	PCAF	HAT A	Human	Mouse, chicken, lizard, zebrafish	H3 (nu)	Coactivator	[70]
	GCN5	HAT A	Human, yeast	Mouse, chicken, lizard, African clawed frog, zebrafish	H2A (nu), H2B (nu/free), H3 (K14), H4 (K8, K16) (nu), Sin1p, All core (nu)	Coactivator	[71]
	HAT1	HAT B	Human	-	H4 (K12, K5) (free)	Acetylation of soluble histones	[72]
	HPA2		Yeast		H3, H4	Chromatin regulator, transferase	[73]
	ELP3			-	H4, H3, H2B, H2A	Transcription elongation	[74]
MYST	MOZ		Human	Mouse, chicken, lizard, zebrafish		Leukemogenesis	[75]
	ESA1	HAT	Yeast		H2A, H3, H4 (free)	Cell cycle progression	[62]
	SAS3 (NuA3)		Yeast		H3	Silencing	[63]
	SAS2		Yeast		Unknown	Silencing	[63]
	HBO1 (KAT7)		Human	Mouse, chicken, lizard, African clawed frog, zebrafish	H3, H4	Origin recognition interaction	[76]
	MOF (KAT8/MSL)		Fruit fly	Mouse, lizard, African clawed frog, zebrafish	H2A, H3, H4	Dosage compensation	[77]
	TIP60 (KAT5)	HAT A	Human	Mouse, chicken, lizard, African clawed frog, zebrafish	H2A, H3, H4	HIV TAT interaction	[78]
P300/CBP	CBP (CREBBP)	HAT A	Humans, worms	-	H4 (K5, 9, 12, 16), all core (nu), p53 (K373, 382, peptide) TFIIF, TFIIEβ	Global coactivator	[79]
	P300	HAT A	-	Mouse, chicken, lizard, zebrafish	-	-	[79]
Basal transcription	TAF1 (TAFII-250)	HAT A	Humans	Mouse, chicken, lizard, African clawed frog, zebrafish	TFIIEβ, H4 (free), H3 (K14)	Transcription initiation	[80]
	TFC3	HAT A	-	-	H2A, H3, H4	-	[81]
	NUT1	HAT A	Yeast		H3, H4	Transcription mediator	[82]
SRC	NCOA3 (SRC-3)	HAT A	Humans	-	H4 (nu), H3	Steroid receptor coactivators	[83]
	NCOA1 (SRC-1)	HAT A	Human	Mouse, chicken, lizard, zebrafish	H4 (nu), H3 (K9, 14, peptide)	Steroid and nuclear hormone coactivator	[83]
	NCOA2 (SRC-2)		-	Mouse, chicken, lizard, African clawed frog, zebrafish		-	[84]
	GRIP1		-	Mouse, chicken, lizard, zebrafish		Trafficking and organization of transmembrane proteins	[85]
	ATF2 (CREB2)		-	Mouse, chicken, lizard, African clawed frog, zebrafish		DNA sequence specific binding activator	[86]

Abbreviations: ATF2 = activating transcription factor-2; CBP/CREBBP = CREB binding protein; CREB2 = CAMP response element binding protein-2; ELP3 = elongator protein-3; ESA1 = essential Sas2-related acetyltransferase-1; GCN5 = general control nonderepressible-5; GNAT = Gcn5-related *N*-acetyltransferases; GRIP1 = glutamate receptor interacting protein-1; HAT1 = histone acetyl transferase-1; HBO1 = human acetylase binding to ORC1; HPA2 = histone and other protein acetyltransferase-2; K = lysine; KAT5 = lysine acetyltransferase-5; KAT7 = lysine acetyltransferase-7; KAT8 = lysine acetyltransferase-8; MOF = males absent on the first protein; MOZ = monocytic leukemia zinc finger; MSL = male-specific lethal; MYST = MOZ, YBF2/SAS3, SAS2 and TIP60 protein; NCOA2 = nuclear receptor coactivator-2; ; NCOA3 = nuclear receptor coactivator-3; nu = nucleus; NuA3 = nucleosomal acetyltransferase of histone H3; NUT1 = negative regulation of URS Two-1; PCAF = P300/CBP-associated factor; SAS2 = something about silencing-2; SAS3 = something about silencing-3; SRC-2 = steroid receptor coactivator-2; SRC-3 = steroid receptor coactivator-3; TAF1 = TATA-box binding protein-associated factor-1; TAT = transactivator of transcription; TFIIEβ = transcription factor II E subunit β; TFC3 = transcription factor class-3; TIP60 = 60 kDa Tat-interactive protein.

**Table 3:** Bromodomain family members and their properties.

Subfamily	Proteins	No. of BRDs	Other Domains	Function	Localization	Reference
I	PCAF	1	PCAF_nGNAT	HAT	Nu	[87]
	GCN5L2	1	PCAF_nGNAT	HAT	Nu	[87]
	FALZ/BPTF	1	WSD, PHD, WHIM1, DDT	Transcription factor	Nu	[88]
	CECR2	1	-	Chromatin remodeler	Nu	[89]
II	BAZ1A	1	PHD, WSD, WHIM1, DDT, WAC_Acf1_DNA_bd	Chromatin remodeler	Nu	[90]
	BRDT	2	CTM, ET	Transcription regulator, chromatin remodeler, spermatogenesis	Nu	[91]
	BRD4	2	CTM, ET	Transcription regulator, chromatin remodeler	Nu	[92]
	BRD3	2	ET	Transcription regulator, erythropoiesis	Nu	[93]
	BRD2	2	ET	Transcription regulator	Nu	[93]
III	PHIP	2	WD40	Insulin signaling	Nu	[94]
	BRWD3	2	WD40	JAK-STAT signaling	Nu, Cyt	[95]
	BAZ1B	1	PHD, WSD, WHIM1, WAC_Acf1_DNA_bd	Spermatogenesis, tyrosine kinase, transcription regulator, chromatin remodeler	Nu	[59,96,97]
	BRD8	2	-	Transcription regulator	Nu	[98,99]
	EP300	1	CREB binding, ZZ, HAT, DUF902, KIX, zf-TAZ	HAT	Nu	[100]
	CREBBP	1	Same as above	HAT	Nu	[100]
	WDR9	2	WD40	Chromatin remodeler	Nu	[101,102]
IV	BRD9	1	DUF3512	Component of SWI/SNF	Nu, Cyt	[103]
	BRD7	1	DUF3512	Transcription regulator	Nu	[104]
	BRPF3	1	PWWP, PHD-like, PHD, EPL1	Same as above	Nu	[105]
	BRPF1	1	Same as above	Same as above	Nu, Cyt	[106]
	BRD1	1	Same as above	Same as above	Nu, Cyt	[107]
	ATAD2B	1	AAA	Same as above	Nu	[108]
	ATAD2	1	AAA	Same as above	Nu	[109,110,111,112]
V	BAZ2B	1	PHD, WSD, WHIM1, DDT, MBD	Unknown	Nu, Cyt	[113]
	BAZ2A	1	Same as above	Transcription repressor	Nu, Cyt	[114]
	TRIM66	1	PHD, zf-B_box	Same as above	Nu	[115]
	TRIM33	1	PHD, zf-B_box, zf-RING	Transcription elongation, ligase (ubiquitin)	Nu	[116,117]
	TRIM24	1	PHD, zf-B_box	Transcription regulator	Nu, Cyt	[118]
	SP110	1	SAND, HSR	Same as above	Nu	[119]
	SP100	1	SAND, HSR, HMG_box	Same as above	Nu, Cyt	[120]
	SP140L	1	PHD, SAND, HSR	Unknown	Nu	[121]
	SP140	1	SAND, HSR	Transcription regulator	Nu, Cyt	[122]
VI	TRIM28	1	zf-B_box, zf-RING	Transcription regulator, ligase (E3 SUMO)	Nu	[123]
	MLL	1	SET, FYRN, PHD-like, PHD, zf-CXXC	Methyltransferase (histone)	Nu	[124,125]
VII	TAF1L	2	zf-CCHC_6, DUF3591, TBP- binding	Transcription initiation	Nu	[126]
	TAF1	2	Same as above	Transcription initiation, p53 transcription regulation	Nu	[127]
	ZMYND11	1	PWWP	Transcription repressor	Nu	[128]
	ZMYND8	1	PWWP, DUF3544	Transcription regulator, DNA damage	Nu	[129]
VIII	SMARCA4	1	SnAC, Helicase_C, SNF2_N, BRK, HAS, QLQ	Chromatin remodeler	Nu	[130]
	SMARCA2	1	Same as above	Chromatin remodeler, splicing	Nu	[131]
	PB1	6	HMG_box, BAH	Chromatin remodeler	Nu, Cyt	[132]
	ASH1L	1	BAH, SET	Methyl transferase	Nu, Cyt	[133]

Abbreviations: AAA = ATPases associated with a variety of cellular activities; ACF = ATP-utilizing chromatin assembly and remodeling factor; ASH1L = (absent, small, or homeotic)-1 like protein; ATAD2 = ATPase family, AAA domain containing 2; ATAD2B = ATPase family, AAA domain containing 2B; *BAH =* bromo-adjacent homology; BAZ1A = BRD adjacent zinc finger-1A; BAZ2A = BRD adjacent zinc finger-2A; BAZ1B = BRD adjacent zinc finger-1B; BAZ2B = BRD adjacent zinc finger-2B; BPTF = bromodomain and PHD domain transcription factor; BRD1 = bromodomain-containing protein 1; BRD2 = bromodomain-containing protein 2; BRD3 = bromodomain-containing protein 3; BRD4 = bromodomain-containing protein 4; BRD7 = bromodomain-containing protein 7; BRD8 = bromodomain-containing protein 8; BRD9 = bromodomain-containing protein 9; BRDT = bromodomain testis associated; BRK = brinker; BRPF3= bromodomain and PHD finger containing 3; BRWD3 = bromodomain and WD repeat domain containing 3; CECR2 = cat eye syndrome chromosome region, candidate 2; CREBBP = CREB binding protein; CTM = carboxy-terminal motif; CXXC = two conserved cysteine-rich clusters; Cyt = cytoplasm; DDT = DNA-binding homeobox and different transcription factors; DUF902 = domain of unknown function-902; EP300 = E1A binding protein P300; EPL1 = enhancer of polycomb-like-1; ET = extra-terminal; FALZ = fetal ALZ-50 clone 1 protein; FYRN = ”FY-rich” domain N-terminal; GCN5L2 = general control of amino acid synthesis protein 5-like 2; HMG box = high mobility group box; HSR = homogeneously-staining region; KIX = interactor of kinase-inducible domain; MBD = methyl-CpG-binding domain; MLL = mixed lineage leukemia; Nu = nucleus; PB1 = polymerase basic protein 1; PCAF = P300/CBP-associated factor; PHD = plant homeodomain; PHIP = PH-interacting protein; PWWP = Pro-Trp-Trp-Pro domain; QLQ = conserved Gln, Leu, Gln containing motif; RING = really interesting new gene; SAND = Sp100, AIRE-1, NucP41/75, DEAF-1; SET = Su(var)3-9, enhancer-of-zeste and trithorax; SMARCA2 = SWI/SNF-related, matrix-associated, actin-dependent regulator of chromatin, subfamily A, member 2; SMARCA4 = SWI/SNF-related, matrix-associated, actin-dependent regulator of chromatin, subfamily A, member 4; SnAC = SNF2 ATP coupling; SP100 = Speckled 100 kDa; SP110 = Speckled 110 kDa; SP140L = SP140 nuclear body protein like; SUMO = small ubiquitin-related modifier; TAF1 = TATA-box binding protein associated factor 1; TAF1L = TATA-box binding protein associated factor 1 like; TRIM24 = tripartite motif containing 24; TRIM28 = tripartite motif containing 28; TRIM33 = tripartite motif containing 33; TRIM66 = tripartite motif containing 66; WAC =WSTF/Acf1/Cbp146; WD40 *=* 40 amino acids motif terminating in tryptophan-aspartic acid (W-D) dipeptide; WDR9 = WD Repeat domain 9; WHIM1 = WSTF, HB1, Itc1p, MBD9 motif 1; WSD = Williams-Beuren Syndrome DDT; zf-CCHC_6 = cysteine- and histidine-rich zinc finger domain; zf-TAZ = transcription adaptor putative zinc finger; ZMYND8 = zinc finger MYND-type containing 8; ZMYND11 = zinc finger MYND-type containing 11; ZZ = two zinc ion binding domain.

**Table 4:** HDAC family members and their properties.

Family	Class	Members	Tissue Distribution	Subcellular Localization	Catalytic Site	Substrates	References
Classic (Zn-dependent)	I	HDAC1	Pervasive	Nucleus	1	STAT3, E2F1, MyoD, p53, SHP, androgen receptor	[149]
		HDAC2	Pervasive	Nucleus	1	STAT3, BCL6, YY1, glucocorticoid receptor	[150]
		HDAC3	Pervasive	Nucleus	1	MEF2D, STAT3, RELA, GATA1, YY1, SHP	[151]
		HDAC8	Pervasive?	Cytoplasm/nucleus	1	-	[151]
	IIA	HDAC4	Brain, skeletal muscle, heart	Cytoplasm/nucleus	1	HP1, GATA1, GCMA	[152]
		HDAC5	Brain, skeletal muscle, heart	Cytoplasm/nucleus	1	HP1, SMAD7, GCMA	[152]
		HDAC7	Placenta, skeletal muscle, heart, pancreas	Cytoplasm/nucleus/mitochondria	1	PLAG2, PLAG1	[153]
		HDAC9	Skeletal muscle, brain	Cytoplasm/nucleus	1	-	[153]
	IIB	HDAC6	Placenta, kidney, liver, heart	Cytoplasm (mostly)	2	SMAD7, SHP, HSP90, α-tubulin	[154]
		HDAC10	Kidney, spleen, liver	Cytoplasm (mostly)	1	-	[151]
	IV	HDAC11	Kidney, skeletal muscle, heart, brain	Cytoplasm/nucleus	2	-	[151]
Modern (NAD^+^-dependent)	III	Mammalian sirtuins (SIRT 1–7)	-	-	-	-	[155]
		Yeast Sir2	-	-	-	-	[155]

Abbreviations: BCL6 = B-cell CLL/lymphoma 6; E2F1 = E2F transcription factor 1; GATA1 = GATA binding protein 1; GCMA = glial cells missing homolog A; HP1 = heterochromatin protein 1; HSP90 = heat shock protein 90 kDa; MEF2D = myocyte enhancer factor 2D; MyoD = myogenic differentiation; PLAG1 = pleomorphic adenoma gene-1; PLAG2 = pleomorphic adenoma gene-2; RELA = V-Rel avian reticuloendotheliosis viral oncogene homolog A; SHP = small heterodimer partner; SIR2 = silent information regulator-2.; SIRT = Sirtuin; SMAD7 = SMAD family member-7; STAT3 = signal transducer and activator of transcription 3; YY1 = Yin and Yang 1.

**Table 5:** Histone methyltransferases (HMTs) and their properties.

Family Name	Complex Member	Substrate	Function	References
Lysine-associated HMTs				
hSET1	HCF1/ASH2/SET1	H3 (K4)	Transcription activation	[185]
MLL4	MENIN, SET1	Same as above	Same as above	[186]
MLL1	Same as above	Same as above	Cell proliferation, transcription activation	[185]
SET7/9		Same as above	Silencing, transcription activation	[187]
SMYD3		Same as above	Same as above	[188]
SET8		H4 (K20)	Heterochromatin, cell cycle	[189]
SUV39H1/2	E2F1/4	H3 (K9)	Heterochromatin, transcription repression	[190]
DOT1L		H3 (K79)	Silencing, transcription activation	[191]
EZH2	EDD-EZH2	H3 (K9, 27)	Silencing, transcription repression	[192]
G9a		Same as above	Same as above	[193]
SETDB1		He (K9)	Silencing, heterochromatin	[194]
Arginine-associated HMTs				
PRMT5	Methylosome	SMN, H4, H2A	Heterochromatin, cell cycle	[195]
PRMT4	NUMAC, P300, NCOA2, PCAF, AR	H3 (R1T, 26), PAB1, CBP, TARP	Transcription coactivation	[196]
PRMT1		H4 (R3), HNRPA2B1, ETOILE, ILE3	Transcription activation	[197]

Abbreviations: AR = androgen receptor; ASH2 = absent, small, or homeotic 2; CBP = CREB binding protein; DOT1L = disruptor of telomeric silencing 1-like; EDD = enriched domain detector; ETIOLE = mouse homolog of T-STAR; EZH2 = enhancer of zeste homolog-2; HCF1 = host cell factor 1; HNRPA2B1 = heterogeneous nuclear ribonucleoproteins A2/B1; MENIN = protein encoded by *men1* gene (multiple endocrine neoplasia type-1); MLL4 = myeloid/lymphoid or mixed-lineage leukemia-4; NCOA2 = nuclear receptor coactivator-2; NUMAC = nucleosomal methylation activator complex; PAB1 = polyadenylate-binding protein-1; PCAF = P300/CBP-associated factor; PRMT = protein arginine N-methyltransferase 5; SET = Su(var)3-9, enhancer-of-zeste and Trithorax; SMYD3 = SET and MYND domain containing-3, TARP = TCR gamma alternate reading frame protein.

**Table 6:** Methyl binding proteins and their properties.

Protein Name	Interaction Partner	Function	References
MeCP2	HDACs, SIN3a	Transcription repression	[229]
	NCOR, c-SKI	Same as above	[246]
	HDAC2, Sin3B	Same as above	[247]
	Methyltransferase (H3K9)	Same as above	[248]
	BRM (SWI/SNF complex)	Same as above	[249]
	HP1	Transcription repression during myogenic differentiation	[250]
	CoREST complex	Neural genes repression	[251]
	HMGB1	Unknown	[252]
	DNMT1	DNA methylation maintenance targeting	[253]
	ATRX	Neural development epigenetic regulation	[254]
	YB-1	Alternative splicing	[255]
	CREB1	Transcription activation	[256]
MBD1	SUV39H1-HP1	Transcription repression	[257]
	MPG	DNA repairing	[258]
	HDAC3, PML-RARα	Silencing	[259]
	CAF-1 p150, SETDB1, MCAF1, MCAF2	Epigenetic inheritance, transcription repression	[260]
MBD2	RFP	Transcription repression enhancement	[261]
	GCNF	Silencing of Oct-4	[262]
	DNMT1	DNA methylation maintenance	[263]
	pCAF, HATs, TACC3	Transcription activation	[264]
	TAX	Same as above	[265]
	SIN3A	Transcription repression	[266]
	MEP50, PRMT5, DOC-1, RbAp46/48, HDAC1/2, P66α/β, MTA1-3, Mi-2 (NuRD complex)	Same as above	[267]
MBD3	GCNF, CDK2AP1	Silencing of Oct-4	[268]
	Dnmt1	DNA methylation maintenance	[263]
	MEP50, PRMT5, DOC-1, RbAp46/48, HDAC1/2, P66α/β, MTA1-3, Mi-2 (NuRD complex)	Transcription repression	[237,269]
MBD4	RFP	Transcription repression enhancement	[261]
	MLH1	DNA repair	[270]
	FADD	Apoptosis	[271]
	HDAC1, SIN3A	Transcription repression	[272]
Kaiso	NCOR	Transcription repression	[273]
	P120	Wnt signaling	[274]
	TCF3	Wnt signaling suppression	[275]

Abbreviations: ATRX = alpha thalassemia/mental retardation syndrome X-linked; BRM = brahma; CAF1 = chromatin assembly factor-1; CoREST = cofactor of repressor element-1 silencing transcription factor; CREB1 = CAMP responsive element binding protein-1; c-SKI = SKI proto-oncogene; DNMT1 = DNA methyl transferase-1; DOC1 = destruction of cyclin B protein-1; FADD = Fas-associated protein with death domain; GCNF = germ cell nuclear factor; HMGB1 = high mobility group box-1; HP1 = heterochromatin protein 1; MCAF1 = MBD1-containing chromatin-associated factor-1; MCAF2 = MBD1-containing chromatin-associated factor-2; MEP50 = methylosome protein-50; MLH1 = MutL homolog-1; MPG = N-methylpurine DNA glycosylase; MTA = metastasis associated; NCOR = nuclear receptor corepressor; NuRD = nucleosome remodeling deacetylase; PML = promyelocytic leukemia; PRMT5 = protein arginine N-methyltransferase-5; RARα = retinoic acid receptor alpha; RbAP 48 = retinoblastoma-binding protein p48; RFP = RET finger protein; SETDB1 = SET domain bifurcated-1; SIN3A = SIN3 transcription regulator homolog A; SIN3B = SIN3 transcription regulator homolog B; SUV39H1 = suppressor of variegation 3–9 homolog-1; TACC3 *=* transforming acidic coiled-coil containing protein-3; TAX = transactivator from the X-gene region; TCF3 = transcription factor-3; YB1 = Y-box binding protein-1.

**Table 7:** Some drugs against HDAC and DNA methylation that are in clinical trials.

Name of Drug	Cancer Type	Phase	Reference
HDAC inhibitors			
Panobinostat	Cutaneous T cell lymphoma	III	[305]
Abexinostat	Follicular lymphoma	II	[306]
Belinostat	Thymic malignancies	II	[307]
Chidamide	Solid lymphomas and tumors	II	[308]
Entinostat	Melanoma	II	[309]
Givinostat	Myeloproliferative neoplasms	II	[310]
Mocetinostat	B-cell malignancies	II	[311]
Practinostat	Prostate cancer	II	[312]
Quisinostat	Cutaneous T cell lymphoma	II	[313]
Valporate	Myelodysplasia	II	[314]
Vorinostat	Acute myeloid leukemia	II	[315]
AR-42	Hematological malignancies	I	[316]
CHR-3996	Solid tumors	I	[317]
Methylation inhibitors			
5-azacytidine	Myelodysplasia	III	[318]
5-aza-2′-deoxycytidine (decitabine)	Myelodysplasia	III	[319]
MG98	Leukemia	II	[320]
5-Fluorodeoxycytidine	Leukemia	I	[321]
Zebularine	Acute myeloid leukemia	Preclinical	[322]
